# ABCG1 Attenuates Oxidative Stress Induced by H_2_O_2_ through the Inhibition of NADPH Oxidase and the Upregulation of Nrf2-Mediated Antioxidant Defense in Endothelial Cells

**DOI:** 10.1155/2020/2095645

**Published:** 2020-12-03

**Authors:** Jiahong Xue, Jiali Fan, Yuan Li, Wenhuan Wu, Qing Yan, Qiangsun Zheng

**Affiliations:** Department of Cardiovascular Medicine, Second Affiliated Hospital of Xi'an Jiaotong University, 157 West Five Road, Xi'an, Shaanxi 710004, China

## Abstract

*Summary*. Oxidative stress is an important factor that is related to endothelial dysfunction. ATP-binding cassette transporter G1 (ABCG1), a regulator of intracellular cholesterol efflux, has been found to prevent endothelial activation in vessel walls. To explore the role of ABCG1 in oxidative stress production in endothelial cells, HUAECs were exposed to H_2_O_2_ and transfected with the specific ABCG1 siRNA or ABCG1 overexpression plasmid. The results showed that overexpression of ABCG1 by ABCG1 plasmid or liver X receptor (LXR) agonist T0901317 treatment inhibited ROS production and MDA content induced by H_2_O_2_ in HUAECs. Furthermore, ABCG1 upregulation blunted the activity of prooxidant NADPH oxidase and the expression of Nox4, one of the NADPH oxidase subunits. Moreover, the increased migration of Nrf2 from the cytoplasm to the nucleus and antioxidant HO-1 expression were detected in HUAECs with upregulation of ABCG1. Conversely, ABCG1 downregulation by ABCG1 siRNA increased NADPH oxidase activity and Nox4 expression and abrogated the increase at Nrf2 nuclear protein levels. In addition, intracellular cholesterol load interfered with the balance between NADPH oxidase activity and HO-1 expression. It was suggested that ABCG1 attenuated oxidative stress induced by H_2_O_2_ in endothelial cells, which might be involved in the balance between decreased NADPH oxidase activity and increased Nrf2/OH-1 antioxidant defense signaling via its regulation for intracellular cholesterol accumulation.

## 1. Introduction

Oxidative stress and reactive oxygen species (ROS) production play a pivotal role in the development of cardiovascular diseases such as atherosclerosis, hypertension, and diabetic cardiovascular complications [1–4]. Oxidative stress is also an important factor that is related to endothelial dysfunction. The mechanism of endogenous sources of ROS includes the mitochondrial respiratory chain, nicotinamide adenine dinucleotide phosphate (NADPH oxidase, NOX), and xanthine oxidase [2, 5]. It has been suggested that the Nox subunits of NADPH oxidase are prominent sources of vascular ROS [6]. On the other hand, the protective role of antioxidant defense, nuclear factor-erythroid 2- (Nrf2-) regulated genes such as heme oxygenase 1 (HO-1), glutathione peroxidase 1 (GPX1), and superoxide dismutase (SOD), fails to scavenge excessive ROS, then leading to oxidative stress accumulation ([3, 7]).

ATP-binding cassette family transporter G1 (ABCG1) is a crucial factor in maintaining sterol and lipid homeostasis by transporting cholesterol efflux to high-density lipoprotein (HDL), which can be regulated by the nuclear receptor family of the transcription factor liver X receptor (LXR) [8]. Previous studies have reported that ABCG1 expression and function are significantly reduced in macrophages and vascular smooth muscle cells, potentially contributing to the foam cell formation of lipid-laden and accelerating atherosclerosis [9, 10]. In addition, ABCG1 is also found to be highly expressed in endothelial cells and likely aids in cholesterol homeostasis to prevent endothelial activation in vessel walls [11–13]. One of the mechanisms is concerned with the suppression of ABCG1 on inflammatory response [12, 14–16]. In addition, Terasaka et al. [12] found that ABCG1 protected against endothelial dysfunction by decreasing ROS production induced by 7-ketocholesterol in a HDL-dependent manner. Similarly, our previous study also found that ABCG1 overexpression reduced ROS production in endothelial cells under high glucose condition [17]. However, the underlying mechanisms of ABCG1 inhibiting the generation of ROS are still elusive.

It has been shown by the study of Tabet et al. [18] that native HDL- and discoidal reconstituted HDL-mediated antioxidant effects and decreased ROS formation under hyperglycemic conditions are dependent on ABCG1. The antioxidant properties of HDL and ABCG1 include the inhibition of NADPH oxidase-dependent ROS generation on the one hand and the upregulation of antioxidant enzymes on the other. To further explore whether ABCG1 expression was able to protect against oxidative stress in endothelial cells and its possible mechanism concerned, overexpression of ABCG1 by a plasmid and downregulation of ABCG1 by siRNA were introduced. The results showed that upregulation of ABCG1 significantly reversed the oxidative stress production induced by H_2_O_2_, which was associated with the balances between prooxidants and antioxidants.

## 2. Methods

### 2.1. Cell Culture

Human umbilical artery endothelial cells (HUAECs, ATCC, Virginia, USA) were cultured in 10% fetal bovine serum (Sigma-Aldrich, St. Louis, USA)/low-glucose Dulbecco's modified Eagle's medium (DMEM; Invitrogen, CA, USA) in a CO_2_/O_2_ incubator at 37°C. Cells were subcultured every 72 h. HUAECs were exposed to H_2_O_2_ (200 *μ*M for 12 h) with or without the LXR agonist T0901317 (5 *μ*g/mL). In addition, HUAECs were transfected with the specific ABCG1 siRNA or ABCG1 overexpression plasmid. Twenty-four hours after transfection, cells were then treated with H_2_O_2_. After the treatment, cell viability was measured with the 3-(4,5-dimethylthiazol-2-yl)-2,5-diphenyl tetrazolium bromide (MTT) assay, showing consistent viability levels of greater than 95%. All experiments were repeated at least three times.

### 2.2. RNA Extraction and Quantitative Real-Time PCR

Total RNA was isolated using the TRIzol reagent (Invitrogen) according to the manufacturer's protocol. First-strand cDNA was synthesized from the total RNA (2 mg) using RevertAid™ First Strand cDNA Synthesis Kit (Fermentas). The mRNA level of the targeted gene was assayed using SYBR Green reagents (Takara, Dalian, China) in a real-time thermocycler (Bio-Rad). The nucleotide sequences of the primers were as follows: Nox4, forward primer 5′-CTGGTGAATGCCCTCAACTT-3′ and reverse primer 5′-GGCCAGGAACAGTTGTGAAG-3′; HO-1, forward primer 5′-AAGACTGCGTTCCTGCTCAAC-3′ and reverse primer 5′-AAAGCCCTACAGCAACTGTCG-3′; and GAPDH, forward primer 5′-TCATCCCTGCCTCTACTG-3′ and reverse primer 5′-TGCTTCACCACCTTCTTG-3′. Levels of Nox4 and HO-1 mRNA were subsequently normalized to GAPDH mRNA levels using the comparative cycle threshold (ΔΔCt) method [19].

### 2.3. Western Blot

The cells were washed with precold PBS three times, and total and fractionated cellular proteins were isolated. The cytoplasmic and nuclear proteins were extracted using the Nuclear and Cytoplasmic Protein Extraction Kit (KeyGEN Biotech). The membrane protein was extracted using the Mem-PER™ Plus Membrane Protein Extraction Kit (Thermo Fisher Scientific) according to the manufacturer's protocol. After protein content determination, extracted proteins were separated by using 10% SDS-PAGE gel and then transferred onto nitrocellulose membranes using a Bio-Rad transfer blotting system. The membranes were incubated with primary antibodies against ABCG1 (Proteintech, USA), Nox4 (Santa Cruz Biotechnology, USA), HO-1 (Santa Cruz Biotechnology, USA), Nrf2 (Cell Signaling Technology), and p47phox (Santa Cruz Biotechnology, USA) antibodies. Proteins were visualized using an enhanced chemiluminescence detection system (ECL, Cell Signaling Technology Inc.). Anti-*β*-actin, anti-lamin B1, anti-Na+/K+ATPase (Santa Cruz Biotechnology, USA) were used to control for equal protein loading.

### 2.4. Detection of Intracellular ROS Production

The generation of intracellular ROS was assessed by incubating cells with the cell-permeable reagent 6-carboxy-2,7-dichlorodihydrofluorescein diacetate, di(acetoxymethyl ester) (CDCFHDA-AM, 20 mM, Sigma-Aldrich) for 20 min at room temperature in the dark as described previously [17]. CDCFH oxidation was measured with excitation at 488 nm and emission at 530 nm using a fluorescence microscope.

### 2.5. Detection of Malondialdehyde (MDA)

MDA, an end product of fatty acid peroxidation, reacts with thiobarbituric acid to form a colored complex that has maximum absorbance at 532 nm [20]. After incubation of HUAECs with H_2_O_2_ for indicated times, cells were harvested with 0.25% trypsin and washed twice with PBS. Then, the contents of MDA were determined using the Lipid Peroxidation MDA Assay Kit (Beyotime Institute of Biotechnology, Changsha, China) according to the manufacturer's instructions.

### 2.6. Measurement of NAD(P)H Oxidase Activity

NADPH oxidase activity was measured using a lucigenin chemiluminescence assay [21]. Briefly, after HUAECs were treated as described above, cells were then washed in ice-cold phosphate-buffered saline, scraped off in lysis buffer (20 mmol/L KH_2_PO_4_, 1 mmol/L EGTA-Na_2_, and protease inhibitors, pH 7.4), transferred to Eppendorf tubes, and sonicated for 3 seconds. The lucigenin-derived chemiluminescence assay was used to determine NAD(P)H oxidase activity in total protein cell homogenates. The reaction was started by the addition of NADPH (0.1 mmol/L) to the suspension (250 *μ*L final volume) containing the sample (50 *μ*L), lucigenin (5 *μ*mol/L), and assay phosphate buffer (50 mmol/L KH_2_PO_4_, 1 mmol/L EGTA-Na_2_, and 150 mmol/L sucrose, pH 7.4). Luminescence was measured every 30 s for 5 min in a luminometer (SpectraMax L, Molecular Devices). After subtraction of the buffer blank, NADPH oxidase activity was expressed as relative light units (RLU)/mg of protein/min.

For another, NADPH oxidase activity was also measured by translocation of cytosolic p47phox to the membrane through western blot.

### 2.7. Measurement of Cellular Lipid Content

Cellular lipids were extracted with chloroform-methanol (2 : 1, *v*/*v*) solution for enzymatic determination of cholesterol. Total intracellular cholesterol was measured by a microenzymatic fluorescence assay as in our previous study [10]. The assay used for total cholesterol evaluation was from Cayman Chemical Co. (Ann Arbor, MI). The fluorescence intensities were measured with a TECAN GENios Pro (excitation 325 nm, emission 415 nm). The cellular protein was determined by the BCA protein assay.

### 2.8. Cholesterol Repletion and Depletion of Endothelial Cells

Cholesterol repletion and depletion of EC with methyl-*β*-cyclodextrin (M*β*CD, Sigma) were performed as described in our previous study [13]. Briefly, HUAECs were incubated with or without prewarmed 10 mM M*β*CD at 37°C for 30 min to deplete cholesterol. HUAECs were then washed with PBS and incubated in the absence or presence of cholesterol (80 *μ*g/mL) and 1.5 mM M*β*CD at 37°C for 1 h to reload HUAECs with cholesterol.

### 2.9. RNA Interference

The following ABCG1 siRNA sequences were designed: forward, 5′-GAGUCUUUCUUCGGGAACATT-3′, and reverse, 5′-UGUUCCCGAAGAAAGACUCTT-3′ (Shanghai GenePharma Co., Ltd.). siRNA against ABCG1 or random siRNA was transfected into HUAECs using the TurboFect siRNA Transfection Reagent (Thermo Scientific, USA) for 24 h [17]. Then, the siRNA-targeted cells were subjected to various treatments.

A predesigned siRNA nucleotide sequence against Nrf-2 was as follows: 5′-UCCCGUUUGUAGAUGACAA-3′ (Invitrogen, Carlsbad, CA, USA). The siRNA for the Nrf-2 gene was transfected into HUAECs with Lipofectamine RNAiMax (Invitrogen, Carlsbad, CA) in accordance with the manufacturer's protocol. After 48 h incubation, the siRNAs of Nrf2 suppressed the expression of Nrf2 proteins by 60% according to western blot analysis.

### 2.10. Plasmid

The OmicsLink™ ORF Expression plasmid of ABCG1 (pReceiver-ABCG1 Expression vector, no. EX-Z0509-M61) and pEZ-M61 with eGFP as the control vector (no. EXEGFP-M61) were obtained from GeneCopoeia™. Plasmid DNA was transfected into HUAECs with the use of a cationic polymer-based TurboFect™ in vitro Transfection Reagent (Thermo Scientific) according to the manufacturer's instructions [17].

### 2.11. Statistical Analysis

The results were reported as means ± standard deviation (SD) of at least three measurements. One-way analysis of variance (ANOVA) was used to compare the means, and the least significant difference (LSD) test when showing statistical significance of differences. Differences were considered significant at *P* ≤ 0.05. All statistical analyses were performed with SPSS19.0.

## 3. Results

### 3.1. Upregulation of ABCG1 Suppressed Oxidative Stress Induced by H_2_O_2_ in HUAECs

**Figure 1:**
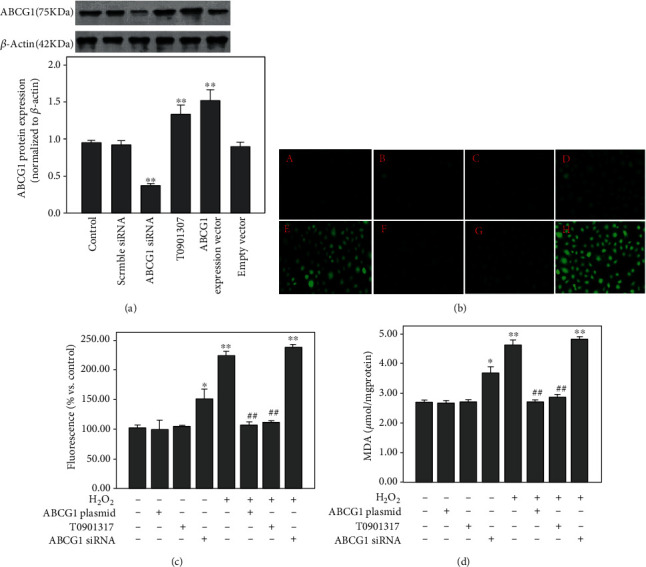
Effect of ABCG1 on H_2_O_2_-induced oxidative stress in HUAECs. HUAECs were transfected with the specific ABCG1 siRNA or ABCG1 overexpression plasmid or preincubated with the liver X receptor (LXR) agonist T0901317 and incubated with 200 *μ*M H_2_O_2_ for 12 h or not. ABCG1 protein levels were determined using(a) western blot assays. The generation of oxidative stress was assessed by the production of (b, c) ROS and (d) MDA. In (b), intracellular ROS production was measured using CDCFHDA-AM fluorescence in different conditions. (A) HUAECs were cultured in medium as normal controls, (B) HUAECs were transfected with the ABCG1 overexpression plasmid, (C) HUAECs were treated with T0901307, (D) HUAECs were transfected with ABCG1 siRNA, (E) HUAECs were exposed for 12 h to 200 *μ*M H_2_O_2_, (F) HUAECs were transfected with the ABCG1 overexpression plasmid and then cultured with H_2_O_2_, (G) HUAECs were pretreated with T0901307 and then cultured with H_2_O_2_, and (H) HUAECs were transfected with ABCG1 siRNA and then cultured with H_2_O_2_. Representative images are shown for three independent experiments (magnification ×100). Data shown are means ± SD (*n* = 3). ^∗^*P* < 0.05, ^∗∗^*P* < 0.001 vs. normal controls; ^##^*P* < 0.001 vs. HUAECs treated with H_2_O_2_ alone.

We first transfected the ABCG1 overexpression plasmid or ABCG1 siRNA into the HUAECs to increase and/or reduce the expression of ABCG1. As shown in Figure 1(a), ABCG1 protein expressions were increased by 57% and/or decreased by 60%, respectively, compared to the endothelial controls.

Furthermore, as indicated in Figure 1, H_2_O_2_ was found to significantly increase ROS generation and MDA content. However, upregulation of ABCG1 expression by either transfecting an ABCG1 expression plasmid into HUAECs or preincubating T0901317 with HUAECs was shown to markedly interfere with the ROS generation (Figures 1(b) and 1(c)) and MDA content (Figure 1(d)). Downregulation of ABCG1 expression by transfecting specific ABCG1 siRNA further increased intracellular ROS production (Figures 1(b) and 1(c)) and MDA content (Figure 1(d)) in H_2_O_2_-treated HUAECs, but not reaching significant differences compared with HUAECs exposed to H_2_O_2_ (*P* = 0.06). In addition, there was statistical difference in ROS generation and MDA content in HUAECs transfected with specific ABCG1 siRNA alone compared with normal controls (*P* < 0.05; Figures 1(c) and 1(d)).

### 3.2. ABCG1 Prevents H_2_O_2_-Induced NADPH Oxidase Activity in HUAECs

**Figure 2:**
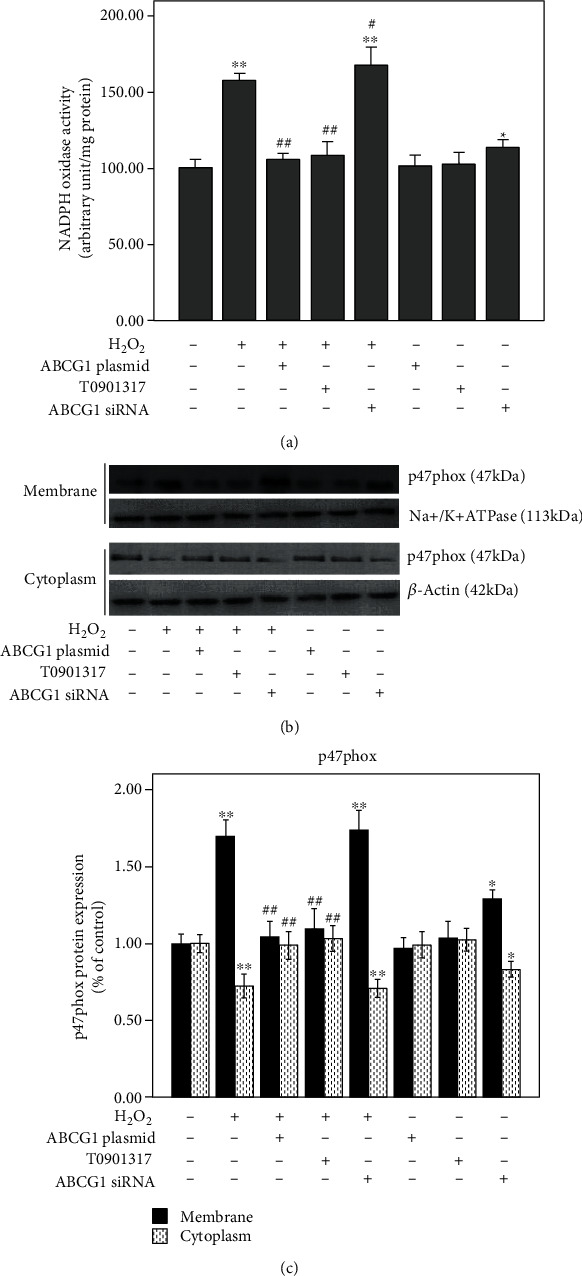
Effect of ABCG1 on H_2_O_2_-induced NADPH oxidase activity in HUAECs. HUAECs were transfected with the specific ABCG1 siRNA or ABCG1 overexpression plasmid or preincubated with T0901317 and then treated with H_2_O_2_ for 12 h or not. At the end of the incubation, NADPH oxidase activity was detected by (a) lucigenin-derived chemiluminescence and by translocation of cytosolic p47phox to the membrane via (b, c) western blotting. Data shown are means ± SD (*n* = 3). ^∗^*P* < 0.05, ^∗∗^*P* < 0.001 vs. normal controls; ^##^*P* < 0.001 vs. HUAECs treated with H_2_O_2_.

It is well known that NADPH oxidase is a prominent source of vascular ROS [6]. To explore the possible mechanisms of ABCG1 inhibiting H_2_O_2_-induced intracellular ROS formation, we focused on NADPH oxidase activity. As shown in Figure 2(a), incubating HUAECs with H_2_O_2_ for 12 h significantly promoted NADPH oxidase activity (*P* < 0.001). However, upregulation of ABCG1 expression by transfecting the ABCG1 expression plasmid or preincubating the LXR agonist T0901317 with HUAECs was observed to obviously reverse the NADPH oxidase activity to the same extent as normal conditions (*P* < 0.001). Conversely, downregulation of ABCG1 expression was found to induce the NADPH oxidase activity whether or not HUAECs were stimulated by H_2_O_2_ (*P* < 0.05).

Furthermore, it has been demonstrated that translocation of cytosolic p47phox to the membrane is an essential process for the activation of the NADPH oxidase [22]. P47phox expression was therefore assessed in membrane and cytosolic protein fractions. As expected, H_2_O_2_ provoked a strong membrane translocation of p47phox, which was attenuated by an increase in ABCG1 expression by both the ABCG1 overexpression plasmid and LXR agonist T0901317 (*P* < 0.001). Conversely, transfection of siRNA targeting ABCG1 increased p47phox translocation in the membrane (*P* < 0.05, Figures 2(b) and 2(c)). However, the level of cytoplasmic P47phox showed concomitantly reverse changes relative to membrane P47phox. These results above suggested a possibility that the protective effect of ABCG1 against oxidative stress, in part, contributed to their ability to block the activation of NADPH oxidase.

### 3.3. ABCG1 Inhibits the Expression of Specific Endothelial NADPH Oxidase Subunits in H_2_O_2_-Treated HUAECs

**Figure 3:**
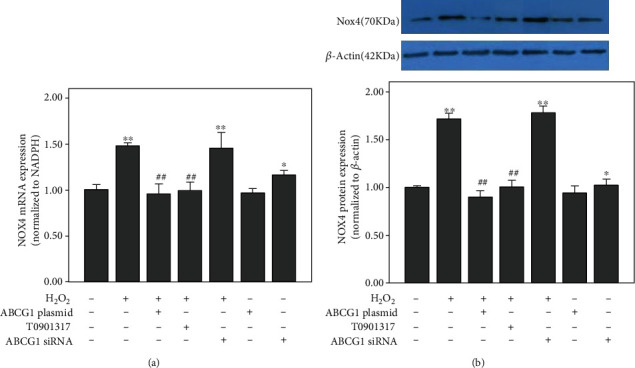
Effect of ABCG1 on H_2_O_2_-induced expression of Nox4 in HUAECs. HUAECs were transfected with the specific ABCG1 siRNA or ABCG1 overexpression plasmid or preincubated with T0901317 and then treated with H_2_O_2_ or not. At the end of the incubation, the expressions of Nox4 (a) mRNA and (b) protein were measured by real-time PCR and western blotting, respectively. Data shown are means ± SD (*n* = 3). ^∗^*P* < 0.05, ^∗∗^*P* < 0.001 vs. controls; ^##^*P* < 0.001 vs. HUAECs incubated with H_2_O_2_.

It has been shown that endothelial cells express all canonical NADPH oxidase subunits including Nox2, Nox4, p22phox, p40phox, p67phox, and p47phox [23]. Nox4 expression is high in endothelial cells, as compared to other Nox isoforms [22, 23]. To evaluate the potential effect of ABCG1 on NADPH oxidase subunits, we analyzed Nox4 expression in HUAECs. As shown in Figure 3, H_2_O_2_ obviously activated Nox4 mRNA and protein levels. Upregulation of ABCG1 by plasmid transfection or by T0901317 treatment blunted the increase in Nox4 mRNA (Figure 3(a)) and protein (Figure 3(b)) levels induced by H_2_O_2_ (*P* < 0.001). Knockdown of ABCG1, by contrast, did not further promote the Nox4 mRNA (Figure 3(a)) and protein (Figure 3(b)) expression under the condition of H_2_O_2_. However, Nox4 expressions were further increased in ABCG1 siRNA alone. Collectively, these results indicated that ABCG1 might be involved in the inhibitory effect on the expression of NADPH oxidase subunits.

### 3.4. ABCG1 Promotes Nrf2-Mediated Antioxidant Redox Response

**Figure 4:**
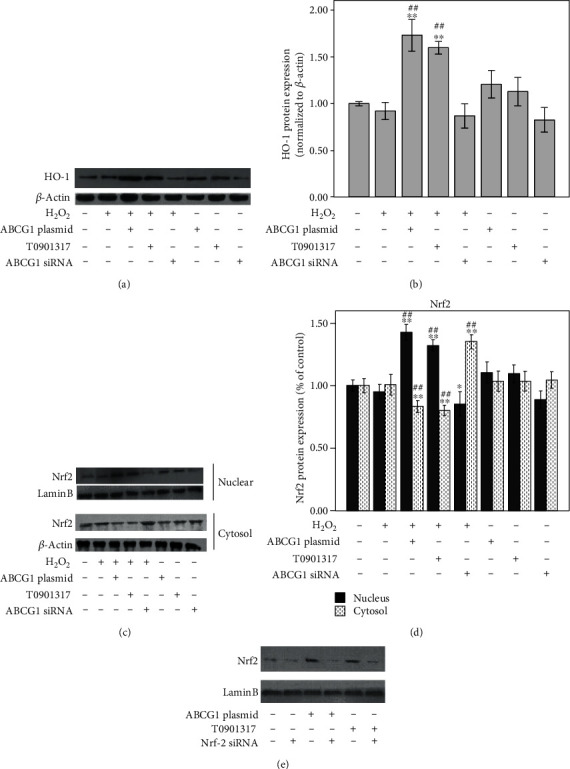
Effect of ABCG1 on antioxidative Nrf2/HO-1 signals in HUAECs treated with H_2_O_2_. HUAECs were transfected with the specific ABCG1 siRNA or ABCG1 overexpression plasmid or preincubated with T0901317 and then treated with H_2_O_2_ or not. (a, b) Antioxidant HO-1 protein expression and (c, d) Nrf2 expression in both the cytoplasm and nucleus were measured by western blotting. (e) Nrf2 expression was inhibited by Nrf2 siRNA under the condition of ABCG1 overexpression. Data shown are means ± SD (*n* = 3). ^∗^*P* < 0.05, ^∗∗^*P* < 0.001 vs. controls; ^##^*P* < 0.001 vs. HUAECs incubated with H_2_O_2_.

Nrf2 is the key antioxidant response regulator gene. To explore if the antioxidative ability of ABCG1 was related to the activation of the Nrf2/HO-1 pathway, we observed the expression of HO-1 and Nrf2. As shown in Figures 4(a) and 4(b), antioxidant HO-1 expression was increased with upregulation of ABCG1, by both ABCG1 plasmid transfection and T0901317 treatment. In addition, the increased migration of Nrf2 from the cytoplasm to the nucleus was also detected in the HUAECs with overexpression of ABCG1. There was a strong nuclear expression of Nrf2, while few expression of Nrf2 in the cytoplasm was simultaneously found in the HUAECs transfected with the ABCG1 plasmid and treated with T0901317. However, nuclear Nrf2 expression was observed to be decreased with ABCG1 silencing (Figures 4(c) and 4(d)).

To further investigate whether Nrf2 knockdown affects the ability of ABCG1 to modulate changes in oxidative stress, transfection of HUAECs with Nrf-2 siRNA was performed. The results showed that transfection with the Nrf2 siRNA for gene knockdown successfully abrogated the ABCG1-induced increase at Nrf2 nuclear protein levels, under the condition of both ABCG1 plasmid transfection and preincubation of HUAECs with T0901317 (Figure 4(e)). Together, these results indicated that the protective effect of ABCG1 on oxidative stress was at least partly through the activation of the Nrf2/HO-1 pathway.

### 3.5. Cholesterol Overload Is Responsible for NADPH Oxidase Activity and HO-1 Expression

**Figure 5:**
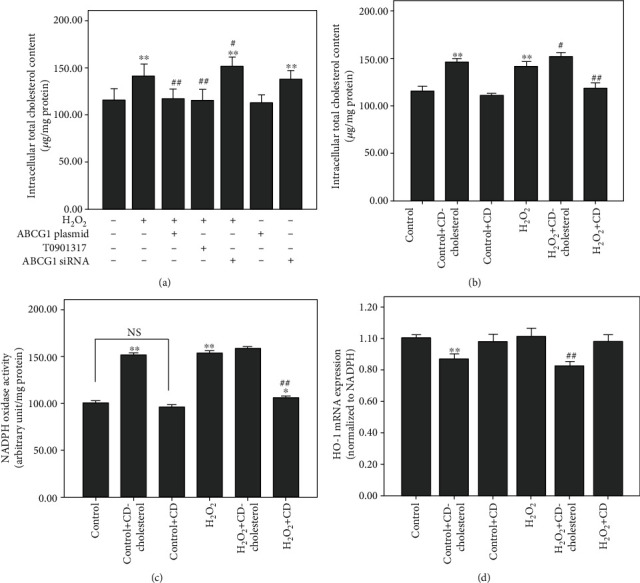
Effect of intracellular cholesterol accumulation on prooxidant NADPH oxidase activity and antioxidant HO-1 expression. (a) HUAECs were transfected with the specific ABCG1 siRNA or ABCG1 overexpression plasmid or preincubated with T0901317 and then treated with H_2_O_2_ or not. Intracellular total cholesterol contents were measured by a microenzymatic fluorescence assay. (b) Intracellular total cholesterol contents were measured after cholesterol repletion or cholesterol depletion using cholesterol-loaded cyclodextrin (CD) or M*β*CD in HUAECs. (c) NADPH oxidase activity was detected after cholesterol repletion or cholesterol depletion in HUAECs. (d) HO-1 mRNA expression was measured by real-time PCR after cholesterol repletion and cholesterol depletion in HUAECs. Data shown are means ± SD (*n* = 3). ^∗^*P* < 0.05 and ^∗∗^*P* < 0.001 vs. controls; ^#^*P* < 0.05 and ^##^*P* < 0.001 vs. HUAECs incubated with H_2_O_2_.

It is well known that ABCG1 is a regulator of cholesterol efflux to HDL and its expression influences the intracellular cholesterol content. As shown in Figure 5(a), we found that H_2_O_2_ increased intracellular total cholesterol content, which could be reversed by upregulating ABCG1 expression by both transfecting the ABCG1 expression plasmid and by preincubating the LXR agonist T0901317 with HUAECs. However, ABCG1 siRNA moderately increased the intracellular cholesterol content in H_2_O_2_-incubated HUAECs. To elucidate whether cellular cholesterol accumulation affects the NADPH oxidase activity and antioxidant HO-1 expression, we loaded control EC with soluble cholesterol using cholesterol-loaded cyclodextrin (CD). As shown in Figure 5(b), CD-cholesterol loading led to a significantly higher amount of total cholesterol content. Cholesterol repletion also resulted in increased NADPH oxidase activity and decreased HO-1 mRNA expression compared to controls (Figures 5(c) and 5(d)). However, cholesterol depletion with free CD reduced intracellular cholesterol accumulation induced by H_2_O_2_ (Figure 5(b)). Meanwhile, NADPH oxidase activity was shown to be reduced, and HO-1 mRNA expression was found to be reversed by cholesterol depletion with free CD in H_2_O_2_-treated HUAECs (Figures 5(c) and 5(d)). The results suggested that cholesterol overload was responsible for the increased activity of NADPH oxidase and decreased antioxidant HO-1 expression, and the effects of ABCG1 on NADPH oxidase activity and the Nrf2/HO-1 pathway were likely associated with its ability to regulate lipid content.

## 4. Discussion

In this study, we showed that upregulation of ABCG1 inhibited oxidative stress induced by H_2_O_2_ in endothelial cells, which was seemly related to the suppression of NADPH oxidase-dependent oxidative stress generation on the one hand and the upregulation of antioxidant redox response of the Nrf2/HO-1 pathway on the other. We found, moreover, that intracellular cholesterol load interfered with the balance between prooxidant NADPH oxidase and antioxidative HO-1, suggesting that the effect of ABCG1 on modulating redox status in endothelial cells was associated with the ability of its regulation for intracellular cholesterol accumulation.

One of the ATP-binding cassette transporter family members, ABCG1, is widely known as a regulator of cellular lipid metabolism [24, 25]. Its function in removing cellular cholesterol from macrophages is well established [8, 24, 25]. ABCG1 also has been shown to be highly expressed in endothelial cells and vascular smooth muscle cells [10–13, 15–17]. A line of studies have found that ABCG1 plays a key role in preserving endothelial function [12, 13, 15–17]. The protective effect of ABCG1 on endothelial function is associated with decreased ROS production regulated by ABCG1-mediated efflux of oxysterols to HDL [12]. Tabet et al. also showed that the antioxidant properties of HDL were dependent on ABCG1, which was related to their ability to efflux cellular cholesterol [18]. Similarly, it has also been shown that deficiency in SR-BI, a regulator of HDL metabolism, results in a significant increase in oxidative stress [26]. Charvet et al. identified a new beneficial role of the ABC transporter in dampening the oxidative burst, which was concerned with the assembly of NADPH oxidase (NOX) 2 complexes [27]. In line with the studies above, the current study found that upregulation of ABCG1 attenuated H_2_O_2_-induced oxidative stress and reduced H_2_O_2_-increased intracellular cholesterol accumulation. Furthermore, it has been known that intracellular cholesterol accumulation is related to the ROS production [28]. Moreover, ROS production inhibits the expression level of ABC transporters [29]. Therefore, it was suggested that the inhibitory effect of ABCG1 on oxidative stress was associated with its regulation for cholesterol efflux.

Oxidative stress is the result of an imbalance between increased generation of reactive oxygen species (ROS) and the decreased ability of endogenous antioxidant systems to scavenge them. It is now well documented that ROS originates from membrane and mitochondrial enzymatic resources [5]. Among the major sources of ROS is the NADPH oxidase, a multi-subunit family of enzymes with each member being distinguished by the specific Nox catalytic subunit, including Nox2, Nox4, p22phox, p40phox,p67phox, p47phox, and the small GTPase Rac ([23]; Sumimoto, 2008; [6]). Among them, Nox4 expression is high in endothelial cells, as compared to other Nox isoforms [22, 23]. Results from the present study suggested that the antioxidant properties of ABCG1 might include the inhibition of Nox4 expression. Furthermore, the reduced NADPH activity coincided with lower Nox4 protein levels that blunted H_2_O_2_-induced oxidative stress in the endothelial cells with overexpression of ABCG1. In contrast, higher ROS generation and MDA levels induced by H_2_O_2_ might be explained by increased NADPH activity and enhanced Nox4 expression in endothelial cells with specific ABCG1 deficiency. The results were in accordance with the study of Tabet et al. [18]; they suggested that HDL, in an ABCG1-dependent fashion, inhibited high glucose-induced redox signaling by the inhibition of Nox2 protein expression and NADPH oxidase activity in human monocyte-derived macrophages. In addition, the activation of the NADPH oxidase is regulated by the translocation of p47phox from the cytoplasm to the plasma membrane [18, 22]. Results from the present study showed decreased membrane p47phox expression compared to increased p47phox expression in the cytoplasm under ABCG1 overexpression condition, further indicating that the decreased oxidation status by ABCG1 overexpression would be most likely attributed to the decreased NADPH subunit protein and activity in the H_2_O_2_-treated endothelial cells.

On the other hand, to protect the cells against ROS, a highly complex antioxidant protection system is involved at the same time. Nuclear factor erythroid 2-related factor 2 (Nrf2) is a transcription factor involved in cellular redox homeostasis. The activity of Nrf2 and the expression of its downstream antioxidant genes, such as HO-1, might serve as a marker of antioxidant response to ROS [3].

Normally, Nrf2 is localized in the cytoplasm primarily through interaction with Kelch-like ECH-associated protein 1 (Keap1). Under the cellular stresses, Nrf2 is released from Keap1 and migrates from the cytoplasm to the nucleus to promote the expression of antioxidant proteins [3, 30]. The current observation was in agreement with earlier findings that migration of Nrf2 from the cytoplasm to the nucleus was in response to the induction of ABCG1. By contrast, few nuclear expressions of Nrf2 and HO-1 were observed under the condition of ABCG1 silencing. Furthermore, increased nuclear expression of Nrf2 by ABCG1 was also offset by Nrf2 siRNA, suggesting the regulation of ABCG1 on antioxidative redox signaling of Nrf2/HO-1. Similarly, antioxidant properties of HDL, in an ABCG1-dependent manner, augmented SOD1 and SOD2 levels under hyperglycemic conditions [18]. It was known that both SOD1 and SOD2 were the target genes of Nrf2 [3]. Furthermore, Nrf2 is also involved in the regulation of ABCG1 and ABCA1 expression [31–33]. Therefore, it was indicated that the inhibitory effect of ABCG1 on oxidative stress might partly contribute to the increased antioxidants of Nrf2 signaling.

The relationship between hypercholesterolemia and oxidative stress has been extensively investigated [28, 34]. Intracellular cholesterol accumulation impaired antioxidant activities and increased superoxide generation from NOX [28, 35]. In agreement with these studies, our results also found that intracellular cholesterol loading led to a significantly increased NADPH oxidase activity and decreased antioxidant HO-1 expression. However, cholesterol depletion in H_2_O_2_-treated endothelial cells reversed both NADPH oxidase activity and HO-1 expression to the same extent as normal conditions. It was well suggested that ABCG1 exhibited a protective effect against H_2_O_2_-induced oxidative stress, which might be closely related to the modulation of cellular lipid content.

## 5. Conclusions

In summary, the results indicated that ABCG1 potently attenuated H_2_O_2_-induced oxidative stress in HUAECs by adjusting the balance between NADPH oxidase-dependent ROS generation and Nrf2-mediated antioxidant defense signaling. Moreover, the underlying mechanisms might involve its regulation for cellular lipid accumulation.

## Data Availability

The raw/processed data required to reproduce these findings cannot be shared at this time as the data also forms part of an ongoing study.
